# Supplementary material for: SARS-CoV-2 infection of human brain microvascular endothelial cells leads to inflammatory activation through NF-κB non-canonical pathway and mitochondrial remodeling
Source: Res Sq. 2022 Jun 16:rs.3.rs-1762855. Preprint. [Version 1] doi: 10.21203/rs.3.rs-1762855/v1 (PMC9216729; doi:10.21203/rs.3.rs-1762855/v1)
Supplement: Supplement 1 [file 12e9dbc049b06a69ecb01da3.docx]

**Table S1:** RT-qPCR primer sequences used in this work

| Gene name | Forward primer (5’-3’) | Reverse Primer (5’-3’) |
| --- | --- | --- |
| ACE2 | TCCATTGGTCTTCTGTCACCCG | AGACCATCCACCTCCACTTCTC |
| Claudin-5 | GTGTCGCAGCAGAAGTACGAG | ACGTGTCCTCCTTGTTTAAG |
| Drp1 | GATGCCATAGTTGAAGTGGTGAC | CCACAAGCATCAGCAAAGTCTGG |
| Fis1 | CAAGGAACTGGAGCGGCTCATT | GGACACAGCAAGTCCGATGAGT |
| HIF-1α | TATGAGCCAGAAGAACTTTTAGGC | CACCTCTTTTGGCAAGCATCCTG |
| MFF | CAA GGT TCC AGG CAC CGA TTT C | GCG ACÇA AAA TGC CAC GAG CAG A |
| Mfn2 | ATT GCA GAG GCG GTT CGA CTC A | TTC AGT CGG TCT TGC CGC TCT T |
| SARS-CoV-2 Spike1 | CTACATGCACCAGCAACTGT | CACCTGTGCCTGTTAAACCA |
| TOMM20 | CGA CCG CAA AAG ACG AAG TGA C | GCT TCA GCA TCT TTA AGG TCA GG |
| ZO- 1 | ACCAGTAAGTCGTCCTGATCC | TCGGCCAAATCTTCTCACTCC |

**Table S2:** Antibodies used in this work

| Target name | Company name | Reference | Host | Dilution |
| --- | --- | --- | --- | --- |
| TOMM20 | Thermo Fisher Scientific | MA5 24859 | Rabbit | 1:200 |
| TOMM20 | Cell Signaling Technologies | PA5-52843 | Rabbit | 1:1,000 |
| ZO-1 | Thermo Fisher Scientific | 33-9100 | Mouse | 1:200 |
| Claudin-5 | Thermo Fisher Scientific | 35-2500 | Mouse | 1:1,000 |
| Cleaved caspase3 | Cell Signaling Technologies | 9661S | Rabbit | 1:200 |
| Spike1 | Thermo Fisher Scientific | 703971 | Rabbit | 1 :100 |
| TMPRSS2 | Thermo Fisher Scientific | PA5-14264 | Rabbit | 1 :1,000 |
| ACE2 | ABCAM | ab15348 | Rabbit | 1 :1,000 |
| MFF | Cell Signaling Technologies | 86668s | Rabbit | 1 :1,000 |
| Fis1 | ABCAM | ab156865 | Rabbit | 1 :1,000 |
| Drp1 | Cell Signaling Technologies | 14647s | Mouse | 1 :1,000 |
| phosphoDrp1 (S616) | Cell Signaling Technologies | #3455 | Rabbit | 1 :1,000 |
| Mitofusin2 (Mfn2) | Cell Signaling Technologies | 11925T | Rabbit | 1 :1,000 |
| AlexaFluor 546 anti-mouse IgG (H+L) | Thermo | A11003 | Goat | 1:1,000 |
| AlexaFluor 488 – anti-rabbit IgG (H+L) | Thermo | A32790 | Donkey | 1:1,000 |
